# Economic analysis of different throughput scenarios and implementation strategies of computer-aided detection software as a screening and triage test for pulmonary TB

**DOI:** 10.1371/journal.pone.0277393

**Published:** 2022-12-30

**Authors:** Saima Bashir, Sandra V. Kik, Morten Ruhwald, Amir Khan, Muhammad Tariq, Hamidah Hussain, Claudia M. Denkinger

**Affiliations:** 1 Division of Tropical Medicine, Center of Infectious Diseases, Heidelberg University Hospital, Heidelberg, Germany; 2 FIND, The Global Alliance for Diagnostics, Geneva, Switzerland; 3 Interactive Research and Development, Global, Singapore; 4 Mercy Corps, Islamabad, Pakistan; Shandong Public Health Clinical Center: Shandong Provincial Chest Hospital, CHINA

## Abstract

**Background:**

Artificial Intelligence (AI) systems have demonstrated potential in detecting tuberculosis (TB) associated abnormalities from chest X-ray (CXR) images. Thus, they might provide a solution to radiologist shortages in high TB burden countries. However, the cost of implementing computer-aided detection (CAD) software has thus far been understudied. In this study, we performed a costing analysis of CAD software when used as a screening or triage test for pulmonary TB, estimated the incremental cost compared to a radiologist reading of different throughput scenarios, and predicted the cost for the national scale-up plan in Pakistan.

**Methods:**

For the study, we focused on CAD software reviewed by the World Health Organization (CAD4TB, Lunit INSIGHT CXR, qXR) or listed in the Global Drug Facility diagnostics catalogue (CAD4TB, InferRead). Costing information was obtained from the CAD software developers. CAD4TB and InferRead use a perpetual license pricing model, while Lunit and qXR are priced per license for restricted number of scans. A major implementer in Pakistan provided costing information for human resource and software training. The per-screen cost was estimated for each CAD software and for radiologist for 1) active case finding, and 2) facility based CXR testing scenarios with throughputs ranging from 50,000–100,000 scans. Moreover, we estimated the scale-up cost for CAD or radiologist CXR reading in Pakistan based on the National Strategic Plan, considering that to reach 80% diagnostic coverage, 50% of TB patients would need to be found through facility-based triage and 30% through active case finding (ACF).

**Results:**

The per-screen cost for CAD4TB (0.25 USD– 2.33 USD) and InferRead (0.19 USD– 2.78 USD) was lower than that of a radiologist (0.70 USD– 0.93 USD) for high throughput scenarios studied. In comparison, the per-screen cost for Lunit (0.94 USD– 1.69 USD) and qXR (0.95 USD—1.9 USD) were only comparable with that of the radiologists in the highest throughput scenario in ACF. To achieve 80 percent diagnostic coverage at scale in Pakistan, the projected additional cost of deploying CAD software to complement the current infrastructure over a four-year period were estimated at 2.65–19.23 million USD, whereas Human readers, would cost an additional 23.97 million USD.

**Conclusions:**

Our findings suggest that using CAD software could enable large-scale screening programs in high TB-burden countries and be less costly than radiologist. To achieve minimum cost, the target number of screens in a specific screening strategy should be carefully considered when selecting CAD software, along with the offered pricing structure and other aspects such as performance and operational features. Integrating CAD software in implementation strategies for case finding could be an economical way to attain the intended programmatic goals.

## Introduction

Tuberculosis (TB) is the leading cause of death worldwide from a single infectious disease, except for the previous two years, when COVID-19 took its place. One of the primary causes for the high death rate is the persistent gap in diagnosis and treatment linkage in the care cascade. According to the World Health Organization (WHO), an estimated 4.1 million out of 9.9 million people went undiagnosed or unreported [1]. This diagnostic gap was further aggravated which was 2.9 million in 2019, through the disruption of TB care services in 2020 due to the COVID-19 pandemic [1,2]. With its End TB Strategy, WHO aims to reduce TB incidence by 90% and TB-related deaths by 95% by 2035 [1]. To achieve these ambitious targets, it is critical to implement strategies to improve early diagnosis and treatment initiation—and systematic TB screening is a key component of these strategies [3,4].

Following WHO endorsement in 2010, the use of Xpert MTB/RIF has become widespread due to its high diagnostic accuracy [5] However, most high TB-burden countries are resource-constrained, and the cost of Xpert testing is relatively high. Using a triage test before referring to a confirmatory test, such as Xpert, could save resources [6]. Chest X-rays (CXR) for screening and triage in TB care have been used for decades and are relatively inexpensive [7]. Prevalence surveys established that CXR can detect 40 to 79% of people with TB who are asymptomatic, potentially reducing transmission through early diagnosis [8,9]. However, in many resource-constrained countries, the use of CXR for TB disease screening and triage is limited due to a lack of radiologists to interpret images and access to X-ray instruments [10,11].

Artificial Intelligence (AI)-based strategies can identify the presence of TB associated abnormalities from CXR images, and could therefore be a promising solution for overcoming the challenges faced by high TB-burden countries with a limited number of radiologists [12–15]. Several computer-aided detection (CAD) software programs for automated reading of TB CXR images are commercially available, with many more in earlier stages of development [15]. CAD software provides a quantitative assessment of radiographic characteristics in the form of abnormality scores, which indicate the probability of TB. Some also report additional information on the presence of other pathologies. As CAD software for TB provides a nearly instantaneous, standardized interpretation, it has the potential to bring CXR interpretation to settings where there are no qualified human readers, reduce radiologists’ workload and fatigue by prioritizing images with abnormal findings, and support in radiologists’ quality control. Based on the diagnostic accuracy of three commercial products, WHO recently recommended the use of CAD software as a screening or triage test in adults as an alternative to human interpreted CXR reading [16]. In addition to the diagnostic accuracy, essential information on operational characteristics, deployment mechanism, machine compatibility, and data sharing and privacy is available thanks to the combined efforts of FIND and the Stop TB Partnership [15,17,18]. One of the big knowledge gaps about CAD software is that there is very limited information about the cost. Only a few studies have been conducted which assessed the cost of a single product (all of which were earlier versions of CAD4TB; CAD4TB v3.07 and v5) in specific settings, considering only a small sample size [19–21]. The incremental cost of adopting these technologies to replace radiologists within existing infrastructure, which is a critical metric for TB programs and implementers to make informed decisions, has so far remained unclear.

With the aim of addressing this knowledge gap, we conducted an incremental costing analysis of different throughput scenarios for using CAD software compared to radiologist CXR interpretation for pulmonary TB when either deployed in an active case finding or facility-based scenario. Furthermore, we estimated the incremental scale-up cost using CAD versus radiologists when implemented at scale in accordance with the Pakistan National Strategic Plan (NSP, 2020–2023).

## Methods

### Computer-aided detection software

We selected four commercially available CE-certified TB-specific CAD software: CAD4TB (Delft Imaging, Netherlands), Lunit INSIGHT CXR (Lunit, South Korea), qXR (Qure.ai, India), and InferRead DR Chest (Infervision, China). Three of these software programs (CAD4TB, qXR, Lunit INSIGHT CXR) were part of WHO’s recent evidence review, for which performance evaluations were done by FIND, the International Organization of Migration, Stop TB partnership and McGill University and informed the WHO screening guideline [16,22–24];therefore they were included in this study. In September 2021, two software programs (CAD4TB and InferRead) were included in the Stop TB Partnership’s Global Drug Facility (GDF) catalogue, which is why InferRead was added to this study. CAD software in the GDF catalogue feature a perpetual license for processing CXR, with no limit on the time duration and number of readings, whereas the other two companies’ pricing strategy is restricted, with a fixed cost per license plus additional costs if a certain number of screenings is exceeded. In addition to online deployment where the AI processing is done in a cloud, all CAD software included in this study can also run on a box or console (called offline) in areas where online implementation is not possible. While in this study we concentrate on the use of CAD software for the purpose of TB screening, it is important to note that some CAD software, such as Lunit INSIGHT CXR, InferRead and qXR, can additionally detect non-TB thoracic radiological findings on a chest x-ray. A detailed overview and pathologies detected by each CAD software can be found in the appendix (S1 Table).

### Study design

This study is divided into two broad components. The first component contains the cost estimations of per screen incremental cost using CAD software compared to a radiologist for different implementation strategies. The second component consider the national scale-up in Pakistan. Both components consider two different implementation strategies: (1) active case finding (ACF) and (2) facility-based triage and compared the cost of CAD to the cost of radiologists reading the CXRs.

### Different implementation strategies

For both implementation strategies: (1) active case finding (ACF) and (2) facility-based triage, the cost for each of the different CAD software programs was estimated when deployed either as an online (referred to as scenario’s 1a and 2a), or offline modality (scenario’s 1b and 2b).

In the ACF scenario, we consider provider-initiated TB screening in a high-risk population. There are different possible forms of ACF, but in Pakistan, our example country for this analysis, mobile vans usually go out into the community and screen about 120 individuals on any given day (inferred from Indus Health Network experience). In the radiologist scenarios, we assumed that one senior and one junior radiologist would be on site to do the instant interpretation of all CXRs taken (using 50% and 100% of their full time equivalent (fte), respectively). To estimate the cost of CAD in the ACF scenario, we assumed that a single screening unit (mobile van) would be equipped with one X-ray instrument and CAD equipment (licensing for online and box and licensing for offline). Moreover, one junior radiologist would be available for remote support (5% of duty time).

The facility-based triaging scenario assumes that patients who are seeking care at a facility because of symptoms or other signs suggestive of TB receive a CXR. Usually, the X-ray machine is used by several different departments and therefore radiologists who do the interpretation of the CXRs do not only read CXR images for TB but also for other diseases or other types of images. We assumed that per facility, there would be one senior and one junior radiologist, who would be spending 10% and 30% of their time, respectively, on reading CXR images from presumptive TB patients. For the use of CAD in the facility-based scenario, we assumed that the radiologists in the facility would be replaced by CAD, but that 5% of time of a junior radiologist would still be needed to respond to any difficult/complicated interpretation.

### Throughput scenarios

We designed a hypothetical study, with four different scenarios defined by different throughput (1a, 1b, 2a, 2b) to assess the variation in per screen cost and to reflect the differences in size between different health facilities or communities: from 10,000 CXRs to 90,000 CXRs in a given period. We assumed one mobile van for ACF or one facility equipped with a digital X-ray machine would perform the CXRs. Dependent on the licensing strategy, one van or one facility would be equipped with one pack which consists of one box (hardware) and one license for perpetual licensing strategy. Whereas, for a volume-based licensing strategy, it would require one box alongside the licensing cost dependent on the throughput for volume-based licensing strategy. For InferRead the minimal equipment to be purchased as per the developer, is a 5-unit pack of hardware and software, regardless of how many instruments are equipped with CAD.

### National scale-up in Pakistan

**Fig 1:**
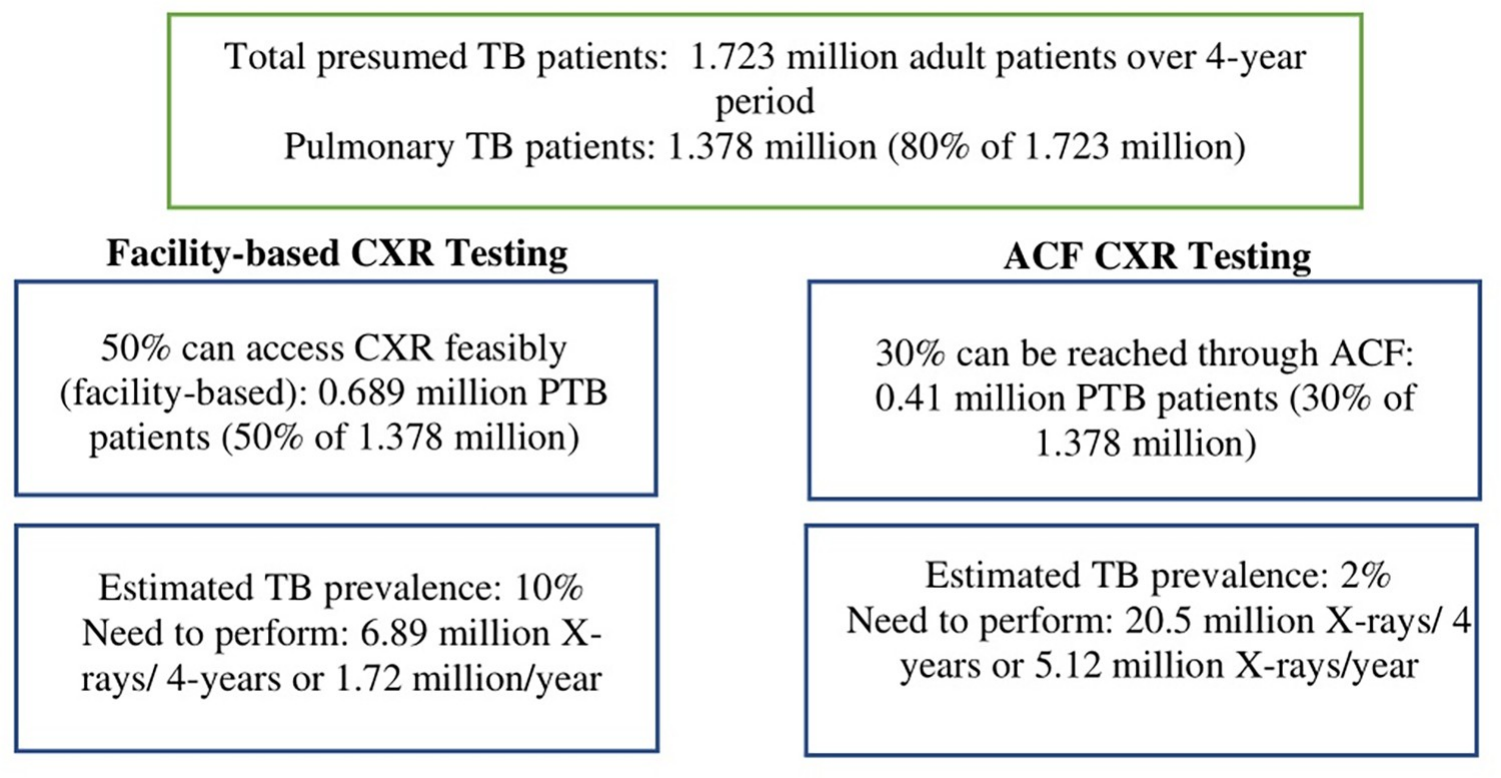
Potentially symptomatic people to be examined with CAD in facility-based and ACF settings to meet Pakistan’s NSP targets (4 years). Active case finding ACF; National Strategic Plan NSP, Computer-aided detection CAD, Pulmonary TBPTB. Facility-based CXR testing is based on the NSP, Pakistan’s assumptions and in addition, we assumed with ACF CXR testing considering the cost with offline deployment of CAD software. The number of presumed TB patients is cumulative for 4 years.

We then estimated the scale-up cost for Pakistan as an example country, comparing the incremental cost of using CAD software to that of using radiologists for CXR interpretation. Pakistan is a high TB-burden and resource-constrained country with a population of 221 million and a TB incidence rate of 230 per 100,000, i.e. a total of 573,000 TB patients per year. Pakistan’s National Strategic Plan (NSP, 2019–2023) aims to diagnose and treat 1.723 million TB patients over four years, with pulmonary TB accounting for 80 percent of all TB patients (1.378 million) [25]. The NSP assumes that half of all pulmonary asymptomatic TB cases. (0.689 million) present to facilities where CXR is feasible, allowing them to be screened with a CXR. Assuming that ten CXRs need to be performed in a facility-based setting to diagnose one symptomatic patient (corresponding to a TB prevalence of 10%), it would be necessary to conduct 6.89 million facility-based CXRs over four years to find all pulmonary TB patients that have access to CXR testing. We inferred from Indus Health Network’s experience that a single facility could perform 40 CXRs per day for presumed TB (11,520/year). Thus, a total of 150 facilities will be required to participate in the testing process to meet the NSP goal (Fig 1). We calculated the per screen cost for the scale up with 150 CAD software packs (hardware, software, installation, and maintenance) for each of the CAD software programs.

In order to achieve the 80% diagnostic coverage, we assumed that in addition to the 50% of TB patients that would be found through facility-based screening, 30% of pulmonary TB patients (0.413 million over 4-years) would be reached through ACF. In the ACF case, we hypothesized that in order to find one pulmonary TB patient, it would be necessary to screen 50 people (independent of symptoms; corresponding to a TB prevalence of 2%) [26,27]. We assumed (inferred from Indus experience) that one van could screen 120 individuals per day (34,560 /year) on average. Thus, a total of 149 vans will be required to find the additional 30% of people with TB through ACF. Currently, Indus and Mercy Corps, which are non-profit organizations supported by Global Fund to Fight AIDS and Tuberculosis and Malaria (GFATM), provide TB care in Pakistan. Their available infrastructure for TB diagnosis in country consists of 64 digital mobile units equipped with CXR devices. An additional 85 digital mobile units will be required to reach the target in four years. The per-screen cost is estimated on the assumption that the offline strategy is the only option used because internet access is not always available.

For sensitivity analyses, we varied the number needed to screen (NNS) in both implementation strategies. We assumed that in order to detect one pulmonary TB patient, instead of 10 (in our main analysis) now 14 presumptive TB patients needed to be tested in the facility-based triage scenario (corresponding to a TB prevalence of 7%), whereas ACF requires (in our main analysis) 100 and 200 presumptive TB patients to be screened, instead of 50, to detect one patient (corresponding to a TB prevalence of 1% and 0.5%).

### Costs

All cost estimates are calculated from a health service provider perspective. We only considered the incremental cost of CAD or radiologists. Equipment other than that required for CAD analysis (e.g., X-ray machine, mobile van, any other facility costs (fixed and recurrent)), and other staff costs beyond radiologists were not included as these costs were considered to be the same irrespective of whether CAD software or radiologists are used for the CXR interpretation (S2 Fig). All the details of cost categories are provided in Table 1.

**Table 1:** Details of cost categories.

Cost Categories	Cost Items	CAD4TB ($)	Infervision ($)	Source	qXR ($)	Lunit ($)	Source (range across vendors, since individual prices could not be disclosed
Equipment cost	CAD box (hardware)	2750	10410 per purchase of 5 boxes (2082/box)	GDF catalogue and Developers	2082–6000	2082–6000	Developers
	software (license)	12750	13500 (2700/lisence)	GDF catalogue and Developers	(0.2–2)/screen	(0.2–2)/screen	Developers
	cloud storage	0.023/GB	0.023/GB	GDF catalogue and Developers	fixed additional cost	fixed proportion of the license cost	Developers
	shipment	Not included	Not included		Not included	Not included	
	remote support and maintenance Extension 1 year	5100	232	GDF catalogue and Developers	Not included	Not included	
	remote support and maintenance Extension 3 year	11475	522	GDF catalogue and Developers	Not included	Not included	
	installation and training	1150	248	GDF catalogue and Developers	Not included	Not included	
	cost for on-site integration with PACS/Workflow	Not included	Not included		Not included	Not included	
	system network charges	Not included	Not included		Not included	Not included	
	bulk purchasing discount	Nil	Nil		possible when purchasing multiple units	possible when purchasing multiple units	Developers
Training cost	Training personnel salaries, venue and catering fees, training materials, and transportation fees						
	Cost for 1 training/van and one facility (3 staff for 2 days)	802	802	IRD	802	802	IRD
Human resources cost	Senior and junior radiologist salaries: We assumed that the additional human resources required for reading X-rays using CAD software are 5% of a junior radiologist’s duty time in both ACF and facility-based screening. In ACF senior and junior radiologists read TB-related X-rays for 50% and 100% of their duty hours, respectively, while facility-based strategies account for 10% and 30% of their duty time, respectively.						
	Salary senior radiologist (1 fte)	2689/month	2689/month	IRD	2689/month	2689/month	IRD
	Salary junior radiologist (1 fte)	1345/month	1345/month	IRD	1345/month	1345/month	IRD

We designed a standard questionnaire to collect cost information from the CAD developers, which is available in the appendix (S2 Table). The questionnaire asked for information on all of the equipment required for the online and offline deployment of the software. Costs for training and human resources were provide by IRD from the ZERO TB initiative implementation in Pakistan in collaboration with the national and provincial TB control programs for both strategies [28]. The ZERO TB project utilized CAD4TB. We extended the assumptions from the project to the use of all other CAD software. The cost data from the developers were in US dollars and data from Pakistan were in Pakistani Rupees. All costs were converted into US dollars using the average exchange rate for the fiscal year (2020–21) by Statistical Bulletin and the State Bank of Pakistan (1 USD = 158 PKR).

## Results

### Estimated cost per screen in two different throughput scenarios

**Fig 2:**
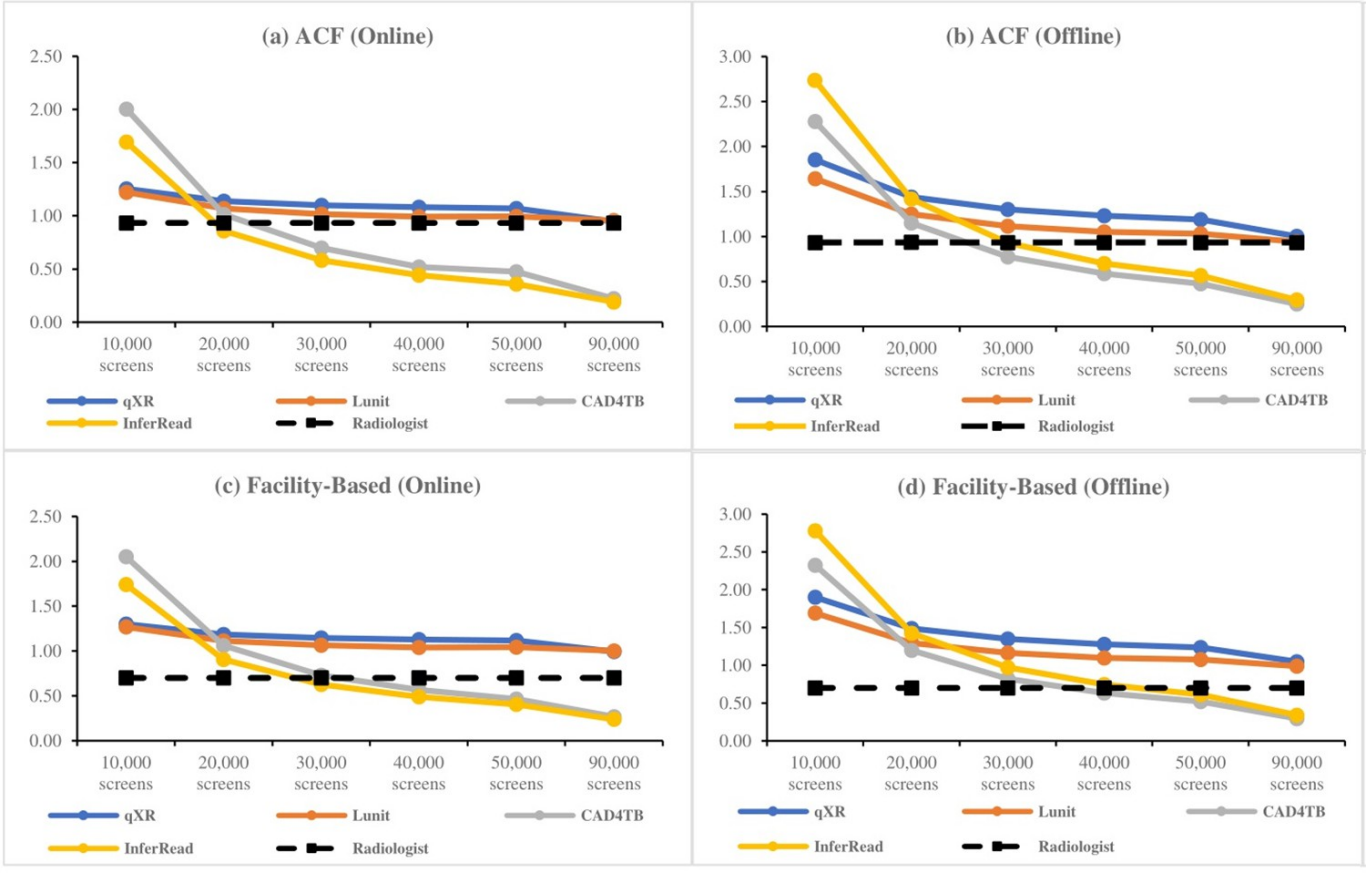
Cost per screen with CAD vs. radiologist for different scenario’s and throughput volumes. Panel (a & b) represents per screen cost with CAD software and radiologist online and offline deployment in ACF implementation strategy and in facility-based triage testing are shown in panel (c &d).

We computed the per-screen cost for all CAD software separately as an ACF (scenario 1) and facility-based test (scenario 2) for pulmonary TB at different levels of throughput with online (1a and 2a) and offline (1b and 2b) CAD deployment as well as the per-screen cost for a human radiologist in these same settings. Results are shown in Fig 2.

In ACF, the estimated per-screen cost with radiologists reading CXR images is 0.93 USD, independent of the throughput; whereas, per-screen cost with CAD software depends on the throughput. The per-screen cost with CAD4TB ranges from 0.25 USD (highest throughput) to 2.28 USD (lowest throughput), from 0.19 USD to 2.73 USD for InferRead, from 0.94 USD to 1.64 USD for Lunit, and from 0.95 USD to 1.85 USD for qXR.

The per-screen costs for the two CAD software programs that had a perpetual licensing costing structure (CAD4TB and InferRead) are considerably lower than the cost with radiologists for high throughput in ACF scenarios. In the case of Lunit and qXR, the per-screen costs for high throughput are comparable to the radiologist costs in ACF.

In facility-based screening, the cost per screen with radiologists reading CXR images is 0.70 USD, irrespective of the throughput. Whereas the cost per screen with CAD4TB ranges from 0.27 USD (highest throughput) to 2.33 (lowest throughput) USD, from 0.24 USD to 2.78 USD for InferRead, from 0.99 USD to 1.69 USD for Lunit, and from 0.99 USD to 1.90 USD for qXR.

Similar to the ACF scenario, the per-screen costs with CAD4TB and InferRead are considerably lower than the cost of the radiologist readings for high throughput in facility-based screening scenarios. Whereas, per screen cost with Lunit and qXR were costlier than the radiologist.

Generally, the per-screen cost with CAD interpretation is lower in the ACF context than that of the same CAD software in the facility-based context and lower for high throughput scenario compared to low throughput scenarios. This is in contrast with radiologists reading CXR images, for which costs were higher in ACF compared to facility-based CXR interpretation and estimated to be constant irrespective of the volume.

The cost difference between different CAD software programs is mainly attributable to the licensing models. Due to the perpetual licensing model, the difference in the per-screen cost between CAD4TB and InferRead and radiologists increases with throughput.

InferRead’s per-screen cost is higher with offline deployment compared to online deployment because offline storage (on the box) is more expensive than the online storage fee charged by the company.

**Fig 3:**
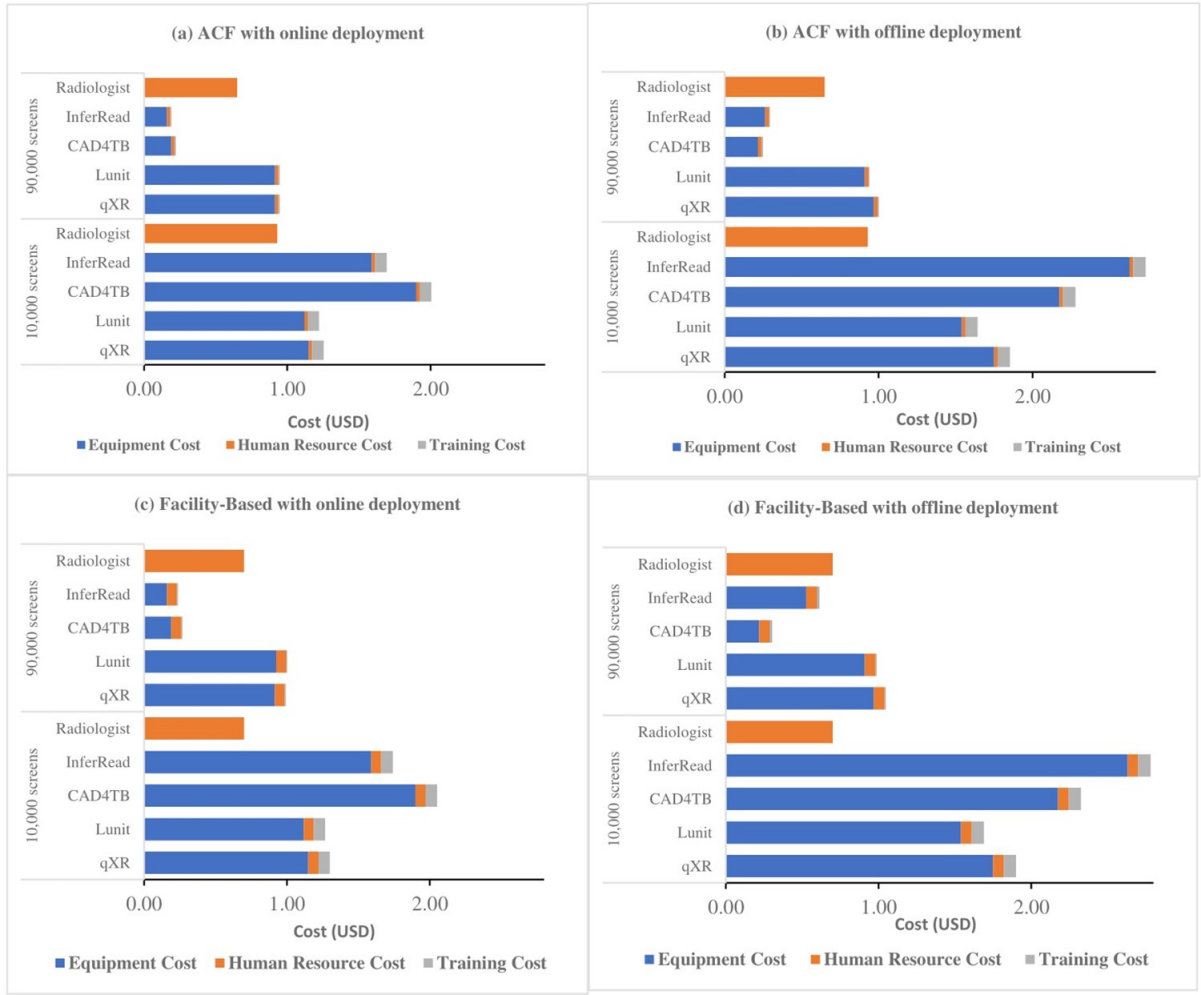
Disaggregated cost per screen with CAD vs. radiologist for different scenarios and throughput volumes.

Fig 3 shows the estimated cost per screen when disaggregated by different cost categories using both implementation strategies with online and offline deployment. The results reveal that the major driver for all CAD software is the equipment cost (including the cost for the hardware, data storage and license fee). The box cost is fixed for all the software programs and ranges from 2,082 USD to 6,000 USD, while the licensing costs depend on the licensing model and range from 2,700 USD to 12,750 USD for software with perpetual licenses. The volume-based licenses start from10,000 USD for 10,000 CXRs and increase with the number of screenings. The data storage cost is driven by volume. Moreover, the average per-screen cost of human resources in ACF and facility-based triage is unaffected by the number of throughputs, whereas the average equipment and training costs per screen go down with an increased number of readings. The detailed results are presented in the appendix (S3 Table).

### Estimated incremental cost of implementation of NSP in Pakistan

We then estimated the expected incremental cost for the national scale-up plan for TB screening according to the NSP using the cost per screen of CAD software and compared this to CXR reading by radiologists.

Table 2 shows the projected incremental cost of scaling up at a national level using the cost per screen detailed above. We first estimated the offline cost per screen in both implementation strategies, considering the hardware and licenses needed to achieve the assumed diagnostic coverage (20.5 million for ACF and 6.9 million CXRs facility-based screening). This changed the per screen cost to 0.23 USD (CAD4TB), 0.06 USD (InferRead), 0.47 USD (Lunit) and 0.66 USD (qXR) for ACF; and for facility-based testing: 0.68 USD (CAD4TB), 0.19 USD (InferRead), 1.11 USD (Lunit), and 0.80 USD (qXR). On the other hand, the per-screen cost with the radiologist remained the same. In the case of CAD software, the licensing strategy is the main driver of differences in per-screen costs. When comparing changes in per-screen cost among CAD software programs, the cost of the perpetually licensed programs drops significantly, as we observed with InferRead, while the cost of CAD4TB is almost identical to the preceding section (for low level of throughput). One of the main reasons behind this, is that the support and maintenance costs multiply as the number of CAD units utilized increase, which is quite high with CAD4TB (11,475 USD per CAD unit with a 3-year extension). The cost for these components are considerably lower for InferRead (522 USD per CAD unit with the 3-year extension). A second reason for the low per screen cost with InferRead is the utilization of all the 5 CAD units in the scale-up scenario, which was higher in the scenarios described above. Overall, the anticipated incremental cost of utilizing InferRead is lowest for all ACF and facility-based X-ray screening in Pakistan over four years (total 2.65 million USD). CAD4TB (9.40 million USD) comes second, and the other CAD software programs have comparable costs (Lunit 17.33 million USD and qXR 19.23 million USD). All CAD software programs are estimated to have lower costs compared to X-ray reading by human reader (23.97 million USD). The cost reduction between the CAD software and radiologist was primarily driven by the difference in per screen cost in ACF implementation (between 5.44 to 17.81 million USD less for CAD software). The cost of ACF with CAD software is substantially lower (ranging from 1.33 to 13.70 million USD for different CAD software for a total of 20.5 million CXRs over 4 years) compared to ACF readings done by radiologists (19.14 million USD). For facility-based implementation, the results are mixed, the incremental cost for InferRead (1.32 million USD) is considerably less than a radiologist, while CAD4TB (4.71 million USD) and qXR (5.53 million USD) are comparable to the cost of a radiologist (4.83 million USD). Lunit carries the highest cost (7.65 million USD). The per-screen cost with Lunit and qXR was similar for the low level of throughput (preceding section). It changed for high throughput due to the shifting licensing pricing structure for different levels of throughput even though both software programs have a volume-based licensing strategy. The second reason is that qXR has a fixed cost for cloud storage, while Lunit’s cost is based on the number of screens, therefore with high volumes, Lunit’s cost increase more than qXR’s. Employing CAD software to achieve 80 percent diagnostic coverage would necessitate an additional investment of between 2.65 to 19.23 million USD, depending on the choice of CAD software. Using human readers, however, would cost an additional 23.97 million USD over the course of four years.

**Table 2:** Expected incremental cost for scale-up at national level.

Implementation strategies	Cost per screen (USD)	Expected cumulative cost for 4 years (million USD)	Expected annual average cost (million USD)
**Radiologist**			
**ACF**	0.93	19.14	4.78
**Facility-based**	0.70	4.83	1.21
**Total**		23.97	5.99
**CAD4TB**			
**ACF**	0.23	4.69	1.17
**Facility-based**	0.68	4.71	1.18
**Total**		9.40	2.35
**InferRead**			
**ACF**	0.06	1.33	0.33
**Facility-based**	0.19	1.32	0.33
**Total**		2.65	0.66
**Lunit**			
**ACF**	0.47	9.68	2.42
**Facility-based**	1.11	7.65	1.91
**Total**		17.33	4.33
**qXR**			
**ACF**	0.66	13.70	3.42
**Facility-based**	0.80	5.53	1.38
**Total**		19.23	4.80

The NSP aims to reach 80% diagnostic coverage and diagnose 1.378 million pulmonary TB patients over a 4-year period. The NSP assumes that 50% of TB patients (n = 0.689 million) will be found through facility-based triage and 30% of TB patients (n = 0.413 million) through active case finding (ACF). For ACF, a TB prevalence of 2% was assumed, meaning that a total of 20.5 million individuals need to be screened with CXRs over a 4-years period. For the facility-based strategy, a TB prevalence of 10% was assumed, meaning that a total of 6.9 million individuals need to be screened with CXR over a 4-year period.

To conduct the required number of CXRs, for the ACF a total of 149 mobile vans equipped with Xray equipment screening are required which on average screen 120 individuals per day. For the facility-based scenario a total 150 facilities with Xray equipment are required which test on average 40 presumptive TB patients per day. Cost included in the per-screen cost with CAD reading are CAD equipment cost (license fee, box, data storage, installation, and maintenance cost), training and human resources (5% of a junior radiologist’s duty time in both implementation strategies) cost. For the per-screen cost with radiologist CXR interpretation we assumed that in ACF senior and junior radiologists read TB-related X-rays for 50% and 100% of their duty hours, respectively, while facility-based strategies account for 10% and 30% of their duty time, respectively (no cost included beyond salary).

### Sensitivity analysis (expected incremental scale-up cost)

We conducted a sensitivity analysis in which we assumed a lower TB prevalence in the testing population. For the ACF we lowered the TB prevalence from 2% to 1% and 0.5% and for the facility-based implementation strategy we lowered the TB prevalence from 10% to 7% (Table 3).

**Table 3:** Sensitivity analysis: Expected incremental cost for scale-up at national level assuming a TB prevalence of 1% and 0.5% in active case finding activities and 7% among individuals screened at facilities.

Implementation strategies	TB prevalence of 1% in active case finding activities and 7% among individuals screened at facilities			TB prevalence of 0.5% in active case finding activities and 7% among individuals screened at facilities		
	Cost per screen (USD)	Expected cumulative cost for 4 years (million USD)	Expected annual average cost (million USD)	Cost per screen (USD)	Expected cumulative cost for 4 years (million USD)	Expected annual average cost (million USD)
**Radiologist**						
**ACF**	0.93	38.29	9.57	0.93	76.58	19.15
**Facility-based**	0.70	6.72	1.68	0.70	6.72	1.68
**Total**		45.01	11.25		83.30	20.83
**CAD4TB**						
**ACF**	0.23	9.37	2.34	0.23	18.70	4.67
**Facility-based**	0.69	6.58	1.65	0.69	6.58	1.65
**Total**		15.95	3.99		25.28	6.32
**InferRead**						
**ACF**	0.06	2.66	0.66	0.07	5.32	1.33
**Facility-based**	0.19	1.85	0.46	0.19	1.85	0.46
**Total**		4.51	1.12		7.17	1.79
**Lunit**						
**ACF**	0.47	19.34	4.83	0.68	55.39	13.98
**Facility-based**	1.13	10.84	2.71	1.13	10.84	2.71
**Total**		30.18	7.54		66.23	16.69
**qXR**						
**ACF**	0.67	27.38	6.85	0.62	51.17	12.79
**Facility-based**	0.74	7.10	1.78	0.74	7.10	1.78
**Total**		34.48	8.63		58.27	14.57

The NSP aims to reach 80% diagnostic coverage and diagnose 1.378 million pulmonary TB patients over a 4-year period. The NSP assumes that 50% of TB patients (n = 0.689 million) will be found through facility-based triage and 30% of TB patients (n = 0.413 million) through active case finding (ACF). In sensitivity analysis, for ACF, a TB prevalence of 1% and 0.5% assumed, meaning that a total of 41 and 82 million individuals respectively need to be screened with CXRs over a 4-years period. For the facility-based strategy, a TB prevalence of 7% was assumed, meaning that a total of 9.6 million need to be screened with CXRs over a 4-year period. To conduct the required number of CXRs, for the ACF a total of 298 and 593 mobile vans respectively equipped with Xray equipment screening are required which on average screen 120 individuals per day. For the facility-based scenario a total 210 facilities with Xray equipment are required which test on average 40 presumptive TB patients per day. Cost included in the per-screen cost with CAD reading are CAD equipment cost (license fee, box, data storage, installation and maintenance cost), training and HR (5% of a junior radiologist’s duty time in both implementation strategies) cost. For per-screen cost with radiologist CXR interpretation we assumed that in ACF senior and junior radiologists read TB-related X-rays for 50% and 100% of their duty hours, respectively, while facility-based strategies account for 10% and 30% of their duty time, respectively (no cost included beyond salary).

According to the sensitivity analysis results, employing CAD software to achieve the targeted diagnostic coverage of 80 percent will require an additional 4.51–34.48 million USD over a 4-year period on top of the current infrastructure, depending on the choice of CAD software. Using human readers, on the other hand, would require 45.01 million USD to cover the total cost for a four-year period. The pattern of results would remain the same if the expected TB prevalence for ACF was further reduced to 0.5%. According to the findings, using CAD software to achieve targeted diagnostic coverage will cost an additional 7.17–66.23 million USD on top of the current infrastructure, depending on the CAD software used. Human readers, on the other hand, would cost 83.30 million USD to cover the total cost over four years.

## Discussion

Several AI-based CAD software programs for automated reading of TB CXR images are currently available on the market [15]. In this study, we compared the incremental costs of four different CAD software for the detection of TB related abnormalities with those for human reader interpretation of CXR images when used in active case finding or facility-based testing strategies in Pakistan.

We showed that the per-screen cost using CAD software with a perpetual license was considerably lower than that of radiologists for ACF and facility-based X-ray testing for high throughput volumes. In case of CAD software with volume-based licensing, the per-screen cost was lower for radiologist at the facilities, while the cost was comparable for high throughput in ACF. The difference in the per-screen cost between CAD software and radiologists increases with increased throughput because the CAD per-screen cost decreases with increased throughput while the cost remains unchanged for radiologists.

Despite the fact that this trend was comparable for all CAD software programs under investigation, the number of screens necessary to attain a lower cost than the radiologist varied. Lunit and qXR reached a comparable cost with the radiologist only for large throughput volumes, whereas CAD4TB and InferRead led to a lower cost per screen. In addition, we identified that the CAD equipment cost was the largest cost component of the CAD per screen cost. For the perpetual licensing model, the hardware and license costs are fixed. For the volume-based licensing model, the data storage and per-screen costs are driven by volume. Installation, support, and maintenance costs are some of the biggest drivers of cost differences for high volume usage of CAD4TB or InferRead. On the other hand, there are no additional installation, support, and maintenance costs for qXR and Lunit. Our findings imply that, in order to minimize cost, the target number of screens in a specific screening strategy should be carefully considered, along with other aspects such as performance and operational features, when selecting a CAD software. Based on the information on testing capacity obtained from IRD, we estimated that in the ACF scenario, one mobile unit could perform the 50,000 CXRs and 100,000 CXRs volumes that we used in our throughput scenarios over roughly 1.5 years or 3 years, respectively. Whereas, in facility-based screening with one X-ray instrument capacitated with CAD, the 50,000 CXRs and 100,000 CXRs would take approximately 4.5 years and 9 years, given that the instrument is also used for other purposes as well.

In the national scale-up analysis, when the expected costs of CAD and radiologists are compared under the most efficient circumstances where vans and equipment are fully utilized, the expected annual cost of radiologist is more than double that for three out of four of the CAD software programs for ACF; however, in facility-based screening, there are mixed findings. We also found that overall, having CXR images read by radiologists would cost about 2.5–10 times more as compared to CAD software to reach the 80% diagnostic coverage at scale over four years. In the case of employing a radiologist, even if we reduce the allocated time of the senior radiologist to read CXR for ACF from 50% to 20%, the estimated radiologist cost remained higher than three out of four CAD software programs. Overall, our findings suggest that existing CAD software, especially when used in large-scale throughput scenarios in both implementation strategies, may be a less costly option for resource constrained countries. Furthermore, considering the often-limited availability of trained radiologists in high TB-burden countries, CAD would be the only option for implementation of CXR for ACF at scale in many settings. The model presented here is for Pakistan; therefore, results and tradeoffs might differ for other high burden countries depending on the resources available.

Most of the previous studies on utilizing CAD for TB focus on diagnostic performance [29,30]. There is limited data on comparative cost analysis associated with TB detection by AI systems and only a few studies [19–21] estimated the cost per test using CAD software as a triage test. One of these showed that reading CXRs with CAD could help reduce the number of higher priced molecular tests required to confirm diagnosis. The cost per screened individual was estimated to be 6.64 USD for CAD4TB—half that of screening an individual directly with a molecular test (13.06 USD). Additionally, the daily throughput was nearly double when CAD4TB was used, compared to the direct molecular screening algorithm [20]. The study estimated that the cost per automated CXR is 1.46 USD using CAD4TB. This cost is higher than in our study since this study did a full costing analysis, including investment costs and the cost of digital X-ray machine, while we only assessed the incremental cost of using CAD or a radiologist for the interpretation of CXR. Moreover, this study was conducted when Delft Imaging was offered on a per-screen basis.

A recent study in a Brazilian prison evaluated screening strategies using different combinations of symptom screening: CXR with CAD4TB interpretation and sputum testing with Xpert [19]. Intriguingly, the study showed that strategies involving CXR were the most expensive and did not increase the total yield when compared to sputum Xpert testing alone. Unfortunately, the study did not include a radiologist-read CXR scenario; however, the study estimated a unit cost of 6.28 USD for CAD4TB. The only additional costing component was the transport cost of the mobile diagnostic unit (0.72 USD), which was not included in our study. The relatively low number of screens (n = 5387) performed in the Brazilian study is likely to explain the relatively high unit cost estimate compared to ours. Another explanation for the high price might be that CAD4TB was not offered with a perpetual license at the time of this study.

Another research study investigated the costs and effects TB-HIV screening in Malawi, using digital CXRs with CAD software (CAD4TBv5) [21]. The study found that digital CXRs interpreted using CAD software were not cost-effective over the trial’s 56-day follow-up period when used in a facility setting and where the tested population had a low prevalence of tuberculosis (around 1%). The authors suggested that CAD has the potential to enhance TB and HIV diagnosis and treatment in a timely and effective manner if these interventions are implemented at a wide scale, and cost and quality advantages can be observed as well. These suggestions are consistent with our findings, as we show cost-savings with both large-scale implementation and increasing prevalence.

Our study has several limitations. We only evaluated the add on cost of the software and digital infrastructure and did not include the costs for the radiology equipment, facility, mobile vans, and support staff as we assumed that this would have been equal for both radiologist and CAD-based strategies. This was done because the focus of this analysis was to compare the incremental cost required using radiologist and CAD software in both implementation strategies. This study was not able to assess the cost effectiveness of the software. According to the literature [23,31], these software programs differ in diagnostic accuracy, which would likely affect the cost effectiveness—something a costing study would not be able to capture. A research study found that the software used to inform the WHO recommendation (qXR, CAD4TB and Lunit) performed significantly better than InferRead, [31]. Another limitation is that the cost data for the different CAD software programs was collected from different sources, and we reconciled the information by consulting with the developers. The developers provided average costs that would be applicable to a country like Pakistan. However, the developers use different pricing schemes for different regions/countries as well as different volume-based pricing structures. Moreover, due to the highly competitive market the pricing structure offered by companies are frequently changing and are negotiable, therefore countries should not take the prices listed here for granted and negotiate prices for their own setting and situation. The costs associated with updates and warranties were not considered for each software program. Further, the costing information used does not take into consideration that some CAD software can additionally detect other non-TB thoracic radiology findings (as described for Lunit, qXR and InferVision), which can provide additional value to the clinical team. We refrained from including this additional potential value because no data exists on the performance of the software solutions for these additional findings (e.g. cardiomegaly). Also this analysis did not take into account that the accuracy of the different CAD software programs, which may impact the cost-effectiveness [22–24,31]. While a comparative analysis of Lunit, qXR and CAD4TB has informed a recent WHO recommendation on surveillance [4] (personal communication C. Denkinger), data on InferRead is limited and suggests a less satisfactory performance [31]. Once reliable comparative data is available across the software solutions, this analysis should be repeated with an effectiveness component to assess cost-effectiveness. Until recently, CAD software costs were not publicly disclosed by developers. With the recent inclusion of two CAD software programs in the GDF catalogue and the results of this study, a start has been made to provide more transparency on the cost of different CAD software for consideration by CAD implementors in decision making.

## Conclusion

CAD software has the potential to enhance TB diagnostic coverage by expanding the CXR reading capacity and augmenting human readers. Previous research has shown that CAD software could outperform human readers, and this study shows that utilizing CAD software for screening might also be a less costly option. Thus, using CAD software integrated into future implementation strategies may be an economically attractive approach to achieve the diagnostic goals.

## Supporting information

S1 Fig Per screen cost with CAD4B and InferRead (suggested licenses) vs. radiologist. (TIF)

S2 Fig Chest radiography (CXR) with computer-aided detection (CAD0 Tools and Radiologist used (I) as a triage test and (II) for systematic screening. (TIF)

S1 Table Overview of the CAD products used. (PDF)

S2 Table Questionnaire. (PDF)

S3 Table Disaggregated cost per screen with CAD vs. radiologist for different level of throughput. (PDF)
