# Supplementary figures and images for: Economic analysis of different throughput scenarios and implementation strategies of computer-aided detection software as a screening and triage test for pulmonary TB
Source: PLoS One. 2022 Dec 30;17(12):e0277393. doi: 10.1371/journal.pone.0277393 (PMC9803287; doi:10.1371/journal.pone.0277393)

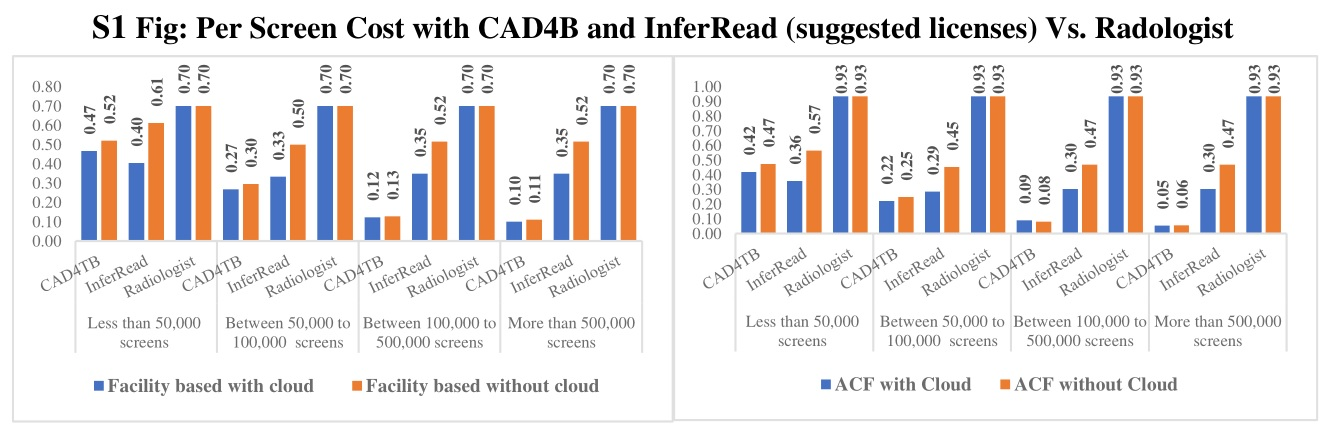

Supplement: S1 Fig — (TIF) [file pone.0277393.s001.tif]

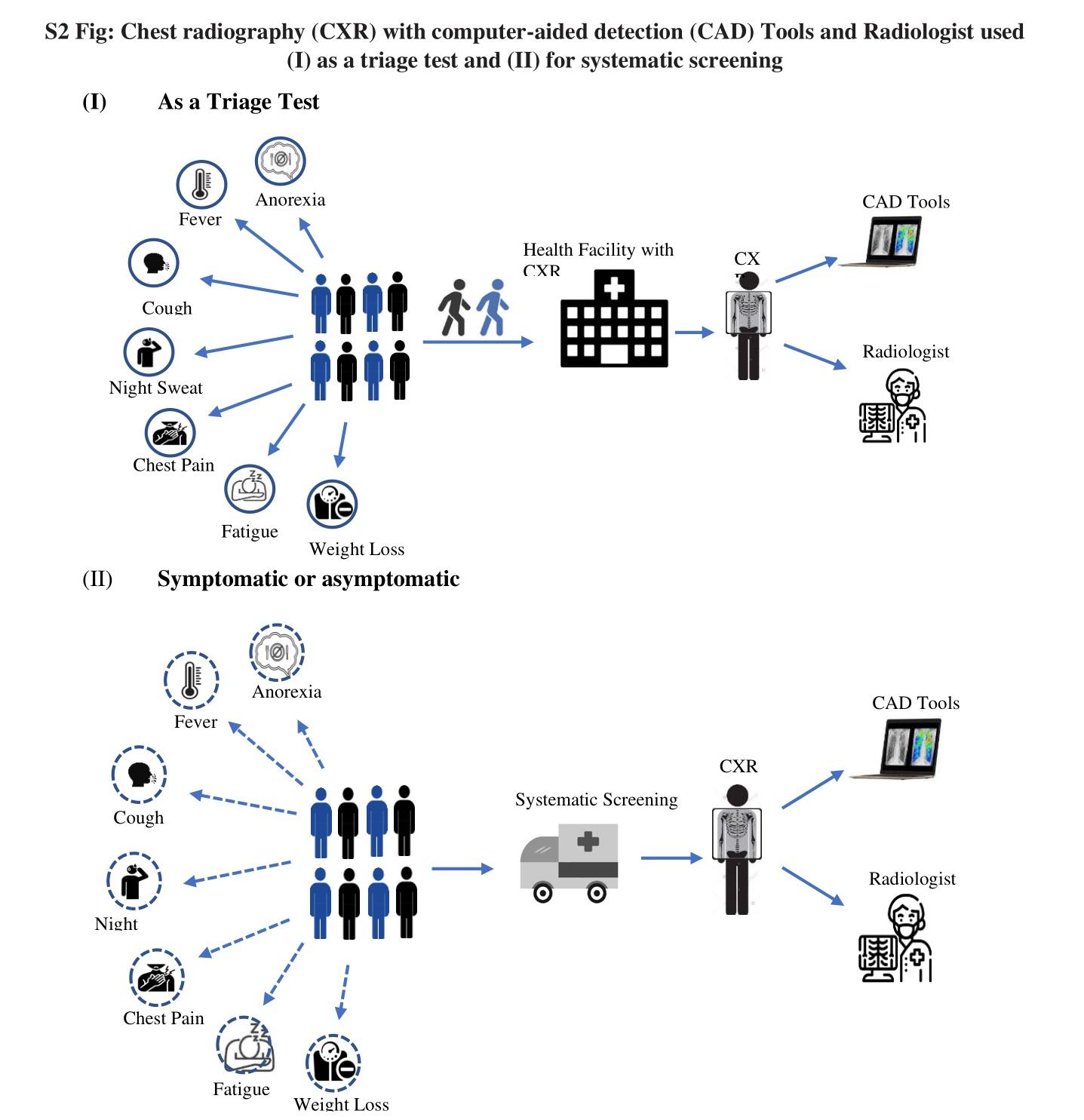

Supplement: S2 Fig — (TIF) [file pone.0277393.s002.tif]
