# Supplementary material for: Economic analysis of different throughput scenarios and implementation strategies of computer-aided detection software as a screening and triage test for pulmonary TB
Source: PLoS One. 2022 Dec 30;17(12):e0277393. doi: 10.1371/journal.pone.0277393 (PMC9803287; doi:10.1371/journal.pone.0277393)
Supplement: S1 Table — (PDF) [file pone.0277393.s003.pdf]

S1 Table: Overview of the CAD Products Used

**Product**	**CAD4TB**	**Lunit INSIGHT**	**qXR**	**InferRead**
Version	Version 6	Version 3.1.0.0	Version 3.0	Version 1.0.0.0
Certification	CE 0344 marked since 2011	CE marked since November 2019	CE marked since April 2018	CE-marked
Intended Age Group	4+ years (regulatory approval)	14+ years (regulatory approval)	6+ years	15+ years
Deployment	Online & offline	Online & offline	Online & offline	Online & offline
Software	CAD4TB 6 runs on Linux. Currently, Ubuntu LTS 18.04 is preferred	CentOS 7.7 or higher<br>Minimum CentOS 7.6	Ubuntu 18.04 is preferred	Ubuntu 18.04 LTS and above
Integration	For version 6, it is possible to send the heatmap and abnormality score in a DICOM format to a picture archiving and communication system (PACS).	Lunit INSIGHT CXR can be integrated with a legacy picture archiving and communication system (PACS) which communicates via DICOM C-Store	qXR can be integrated the product with the client's legacy Picture<br>Archiving and Communication System (PACS)	InferRead can be integrate the product with the client's legacy picture archiving and communication system (PACS)
Processing Time	Less than 20 seconds	About 20 seconds per one X-ray	Less than a minute	less than 5 seconds
Image Input	**Image format:** DICOM from any kind of CXR machine<br>**Image type:** posterior-anterior	**Image format:** DICOM from any kind of CXR machine<br>**Image type:** posterior-anterior chest X-ray, anterior-posterior chest Xray, portable	**Image format:** JPEG, PNG, DICOM from any kind of chest X-ray machine<br>**Image type:** posterior-anterior chest X-ray, anterior-posterior chest Xray, portable	**Image Format**: JPEG, PNG, DICOM<br>**Image type:** posterior-anterior chest X-ray, anterior-posterior chest X-ray
Image Output	*Heat map<br>*Probability score for TB	*Heat map,<br>*Probability score as well as dichotomous output indicating whether each abnormality is present or absent,<br>*Probability score as well as dichotomous output indicating whether TB is likely present or likely absent<br>*Specification of the location of each abnormality	*Probability score as well as dichotomous output indicating whether each abnormality is present or absent.<br>*Probability score for TB as well as dichotomous output indicating whether TB is likely present or likely absent.<br>*A box indicating the location of the abnormalities	* Heat map,<br>*Dichotomous output indicating the presence or absence of the following abnormalities: TB, abscess, airfluid level, atelectasis, blunted costophrenic angle, bronchiectasis, calcification, cavity, chest wall invasion/destruction etc.<br><br>*Probability score for TB,<br>*Probability score for each abnormality,<br>*Location of each abnormality.
Scores	Abnormality score for TB, ranging between 0-100	Abnormality score for TB, ranging between 0-1<br>Default threshold probability score is set at 15% for all abnormalities including TB.<br>Chest abnormalities detected by the product for which a separate<br>abnormality score is given include: atelectasis, calcification, cardiomegaly, consolidation, fibrosis, mediastinal widening, nodule, pleural effusion, pneumoperitoneum, pneumothorax and TB	Abnormality score for TB, ranging between 0-1<br>Abnormalities detected by the product for which a separate abnormality score is given include: abnormal, tuberculosis, opacities (atelectasis, cavities,<br>calcification, consolidation, fibrosis, nodules, reticulo-nodular pattern),<br>emphysema/ hyperinflation, pleural effusion, blunted costophrenic angle, pneumothorax, cardiomegaly, tracheal shift, degenerative spine changes, scoliosis, hilar prominence, rib fractures, COVID-19, pneumoperitoneum, mediastinal widening, elevated hemidiaphragm, abnormal diaphragm shape, lines and tubes (4 types).	Dichotomous output
