# Supplementary material for: Economic analysis of different throughput scenarios and implementation strategies of computer-aided detection software as a screening and triage test for pulmonary TB
Source: PLoS One. 2022 Dec 30;17(12):e0277393. doi: 10.1371/journal.pone.0277393 (PMC9803287; doi:10.1371/journal.pone.0277393)
Supplement: S2 Table — (PDF) [file pone.0277393.s004.pdf]

S2 Table: Questionnaire

Implementation Scenarios		Costing Equipment (hardware+software)	less than 50,000 x-rays per year	between 50,000 to 100,000 x-rays per year	between 100,000 to 500,000 x-rays per year	More than 500,000 x-rays per year
1. Active Outreach	Active screening with cloud storage	Upfront cost for cloud service (cloud storage) including data hosting to be used in a mobile vehicle				
		Is the provided packages are inclusive of network cost? If no, please provide the network cost.				
		Cost of CAD Bundle (Tuberculosis detection software license with x-ray reading).				
		Number of mobile vans that can use the same cloud service (as long as you don't exceed the max number of Xray images).				
		Any Additional cost				
	Active screening without cloud storage	Upfront cost for a CAD box				
		Discount when purchasing a multitude of boxes?				
		Cost of CAD Bundle (Tuberculosis detection software license with x-ray reading)				
		Any Additional cost				
	Cost of Verification Report CAD					
2. Hospital based triage	Static facility with cloud storage	Upfront cost for cloud service (Cloud storage) including data hosting to be used in a static facility				
		Cost of CAD Bundle (Tuberculosis detection software license with x-ray reading)				
		Is the provided packages are inclusive of network cost? If no, please provide the network cost.				
		Number of facilities that can use the same cloud service (as long as you don't exceed the max number of Xray images).				
		Cost for integration with existing PACS/Workflow systems on site?				
		Any Additional cost				
	Static facility without cloud storage	Cost of CAD Bundle (Tuberculosis detection software license with x-ray reading				
		Upfront cost for a CAD box				
		Discount when purchasing a multitude of boxes?				
		Any Additional cost				
	Cost of Verification Report CAD					
**Note: The row of additional cost is added in each of the scenario in case we are missing some important head**						
