# Supplementary material for: Economic analysis of different throughput scenarios and implementation strategies of computer-aided detection software as a screening and triage test for pulmonary TB
Source: PLoS One. 2022 Dec 30;17(12):e0277393. doi: 10.1371/journal.pone.0277393 (PMC9803287; doi:10.1371/journal.pone.0277393)
Supplement: S3 Table — (PDF) [file pone.0277393.s005.pdf]

S3 Table: Disaggregated Cost per Screen with CAD Vs. Radiologist for Different Level of Throughput

Implementation Scenario	Cost Items	qXR	Lunit	CAD4TB	InferRead	Rad	qXR	Lunit	CAD4TB	InferRead	Rad	qXR	Lunit	CAD4TB	InferRead	Rad
		10,000 CXRs					20,000 CXRs					30,000 CXRs				
Active Outreach with Cloud	Equipment Cost	1.15	1.12	1.90	1.59		1.08	1.01	0.95	0.80		1.05	0.97	0.63	0.53	
	Human Resource Cost	0.02	0.02	0.02	0.02	0.93	0.02	0.02	0.02	0.02	0.93	0.02	0.02	0.02	0.02	0.93
	Training Cost	0.08	0.08	0.08	0.08		0.04	0.04	0.04	0.04		0.03	0.03	0.04	0.03	
	Total	1.25	1.22	2.00	1.69	0.93	1.14	1.07	1.01	0.86	0.93	1.10	1.02	0.70	0.58	0.93
Active Outreach without Cloud	Equipment Cost	1.75	1.54	2.18	2.63		1.38	1.19	1.09	1.32		1.25	1.07	0.73	0.88	
	Human Resource Cost	0.02	0.02	0.02	0.02	0.93	0.02	0.02	0.02	0.02	0.93	0.02	0.02	0.02	0.02	0.93
	Training Cost	0.08	0.08	0.08	0.08		0.04	0.04	0.04	0.08		0.03	0.03	0.03	0.03	
	Total	1.85	1.64	2.28	2.73	0.93	1.44	1.25	1.15	1.42	0.93	1.30	1.12	0.78	0.93	0.93
Facility Based with Cloud	Equipment Cost	1.15	1.12	1.90	1.59		1.08	1.01	0.95	0.80		1.05	0.97	0.63	0.53	
	Human Resource Cost	0.07	0.07	0.07	0.07	0.70	0.07	0.07	0.07	0.07	0.70	0.07	0.07	0.07	0.07	0.70
	Training Cost	0.08	0.08	0.08	0.08		0.04	0.04	0.04	0.04		0.03	0.03	0.03	0.03	
	Total	1.30	1.27	2.05	1.74	0.70	1.19	1.12	1.06	0.91	0.70	1.15	1.06	0.73	0.63	0.70
Facility Based without Cloud	Equipment Cost	1.75	1.54	2.18	2.63		1.38	1.19	1.09	1.32		1.25	1.07	0.73	0.88	
	Human Resource Cost	0.07	0.07	0.07	0.07	0.70	0.07	0.07	0.07	0.07	0.70	0.07	0.07	0.07	0.07	0.70
	Training Cost	0.08	0.08	0.08	0.08		0.04	0.04	0.04	0.04		0.03	0.03	0.03	0.03	
	Total	1.90	1.69	2.33	2.78	0.70	1.49	1.30	1.20	1.43	0.70	1.35	1.16	0.82	0.97	0.70
Implementation Scenario	Cost Items	40,000 CXRs					50,000 Screens					90,000 Screens				
Active Outreach with Cloud	Equipment Cost	1.04	0.95	0.48	0.40		1.03	0.96	0.44	0.32		0.92	0.92	0.25	0.16	
	Human Resource Cost	0.02	0.02	0.02	0.02	0.93	0.02	0.02	0.02	0.02	0.93	0.02	0.02	0.02	0.02	0.93
	Training Cost	0.02	0.02	0.02	0.02		0.02	0.02	0.02	0.02		0.008	0.008	0.008	0.008	
	Total	1.08	0.99	0.52	0.44	0.93	1.07	1.00	0.47	0.36	0.93	0.95	0.96	0.29	0.19	0.93
Active Outreach without Cloud	Equipment Cost	1.19	1.01	0.54	0.66		1.15	0.99	0.44	0.53		0.97	0.91	0.22	0.26	
	Human Resource Cost	0.02	0.02	0.02	0.02	0.93	0.02	0.02	0.02	0.02	0.93	0.02	0.02	0.02	0.02	0.93
	Training Cost	0.02	0.02	0.02	0.02		0.02	0.02	0.02	0.02		0.008	0.008	0.008	0.008	
	Total	1.23	1.05	0.59	0.70	0.93	1.19	1.03	0.47	0.57	0.93	1.00	0.94	0.25	0.29	0.93
Facility Based with Cloud	Equipment Cost	1.04	0.95	0.48	0.40		1.03	0.96	0.38	0.32		0.92	0.92	0.19	0.16	
	Human Resource Cost	0.07	0.07	0.07	0.07	0.70	0.07	0.07	0.07	0.07	0.70	0.07	0.07	0.07	0.07	0.70
	Training Cost	0.02	0.02	0.02	0.02		0.02	0.02	0.02	0.02		0.008	0.008	0.008	0.008	
	Total	1.13	1.04	0.57	0.49	0.70	1.12	1.04	0.47	0.40	0.70	0.99	1.00	0.27	0.24	0.70
Facility Based without Cloud	Equipment Cost	1.19	1.01	0.54	0.66		1.15	0.99	0.44	0.53		0.97	0.91	0.22	0.53	
	Human Resource Cost	0.07	0.07	0.07	0.07	0.70	0.07	0.07	0.07	0.07	0.70	0.07	0.07	0.07	0.07	0.70
	Training Cost	0.02	0.02	0.02	0.02		0.02	0.02	0.02	0.02		0.008	0.008	0.02	0.02	
	Total	1.28	1.10	0.63	0.75	0.70	1.24	1.08	0.52	0.61	0.70	1.05	0.99	0.30	0.61	0.70
